# Advances in the Control of Plant Fungal Pathogens

**DOI:** 10.3390/jof12050331

**Published:** 2026-05-02

**Authors:** Paloma Sánchez-Torres

**Affiliations:** Food Biotechnology Department, Consejo Superior de Investigaciones Científicas (CSIC), Instituto de Agroquímica y Tecnología de Alimentos (IATA), Catedrático Agustín Escardino Benlloch 7, Paterna, 46980 Valencia, Spain; psanchez@iata.csic.es

**Keywords:** control, fungal plant pathogens, plant compounds, biocontrol, RNAi, nanotechnology

## Abstract

Fungi are the main causative agents of plant diseases and are responsible for substantial and recurrent damage to agricultural systems. Their activity causes significant reductions in crop productivity and food quality, ultimately contributing to plant deterioration and economic losses. It is estimated that phytopathogenic fungi can compromise up to 30% of global agricultural production. To mitigate microbial deterioration, a wide range of control strategies have been employed, with chemical fungicides being one of the most widely used interventions. However, current approaches to fungal control are rapidly transforming owing to the growing prevalence of fungicide resistance, increasingly stringent regulatory frameworks governing chemical applications, and evolving market demands. Taken together, these factors impose new constraints and drive the development of more sustainable alternative options for effective food control. This review examines the diverse strategies used to control fungal diseases in plants, emphasizing advances in biocontrol agents and biofungicides, as well as emerging tools in the molecular biology, genomics, and biotechnology fields. The aim is to highlight recent developments and prospects that can be integrated into comprehensive disease-management approaches.

## 1. Introduction

Pathogenic fungi remain one of the most destructive threats to global agricultural production, causing severe yield losses and undermining food security and the long-term sustainability of cropping systems. Their impact is intensified by environmental conditions that are increasingly favorable for disease development [1,2], driven by climate change, intensification of agricultural practices, rapid emergence of highly virulent lineages, and widespread evolution of fungicide-resistant populations [3]. Consequently, the effective management of fungal diseases has become a critical challenge in contemporary crop protection.

Recent advances in molecular biology, genomics, biotechnology, and integrated disease management have substantially deepened our understanding of host–pathogen interactions and opened new possibilities for disease control [4]. Innovations such as high-resolution diagnostic tools, genome-editing platforms, improved biocontrol agents, and predictive epidemiological modeling are transforming current approaches to fungal disease management [5].

These advances offer promising alternatives to conventional fungicides, whose effectiveness is increasingly compromised by the evolution of resistance and tightening of regulatory frameworks [6,7].

This review summarizes the main scientific and technological advances in the control of phytopathogenic fungi, emphasizing emerging strategies aimed at improving disease suppression and minimizing environmental impacts. By integrating knowledge from molecular research, ecological principles, and applied plant pathology, we describe the current state of the field and discuss future directions for the sustainable control of fungal diseases.

## 2. Improved Biocontrol Agents

Currently, biocontrol approaches have gained greater relevance as sustainable alternatives to conventional chemical treatments because of their environmental compatibility and lower risk of generating resistance. Key approaches in this context include the use of antagonistic microorganisms, such as fungi, yeasts, bacteria, and viruses [8].

Among the various biological control options, microbial antagonists have stood out for their multifunctional resistance mechanisms against pathogens, adaptability, environmental safety, wide availability, and ability to quickly achieve high yields. These microorganisms are generally found on the surfaces of fruits and vegetables, as well as in plant tissues, roots, and soil. The main groups of microbial antagonists include fungi, yeasts, bacteria, and viruses. Their mechanisms of action include direct interactions with pathogenic fungi through processes such as parasitism, antibiotic synthesis, and competition for nutrients and space. They can also act indirectly by inducing host resistance [9,10].

### 2.1. Filamentous Fungi Biological Control Agents

Fungal biocontrol agents represent an important group of biological antagonists capable of suppressing phytopathogenic fungi through various mechanisms, such as competition for nutrients and space, mycoparasitism, antibiosis, and activation of host defense responses [11]. Among them, Trichoderma species are notable for their rapid growth, strong competitive ability, and production of a wide range of hydrolytic enzymes and antifungal secondary metabolites. Species such as *T. harzianum*, *T. atroviride*, and *T. virens* have demonstrated broad-spectrum efficacy against numerous pre- and post-harvest pathogens [12]. *Trichoderma* can colonize the rhizosphere and outcompete soilborne pathogens, while its enzymatic arsenal—particularly chitinases, glucanases, and proteases—enables the degradation of fungal cell walls in pathogens such as *Fusarium*, *Rhizoctonia*, and *Sclerotinia* [13]

**Figure 1:**
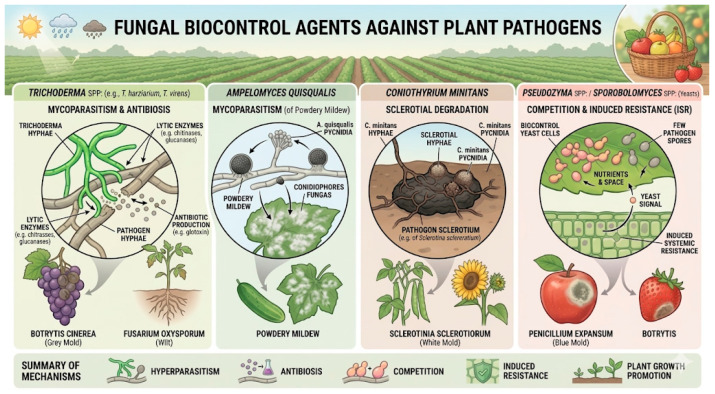
Illustration of various fungal species capable of acting as biocontrol agents against fungal pathogens that affect plants and fruits and the control mechanisms in which they may be involved. Arrows indicate crops affected.

Other fungal taxa, such as *Aureobasidium*, *Clonostachys*, and certain non-pathogenic strains of *Fusarium* and *Penicillium*, have also shown promising biocontrol potential [14,15,16]. These organisms can colonize plant surfaces or rhizospheres, establish stable ecological interactions, and modulate microbial communities to prevent the establishment of pathogens (Figure 1). Their ability to adapt to diverse environmental conditions and persist in plant tissues makes them valuable components of integrated disease management programs [17,18,19].

Despite their potential, the performance of fungal biocontrol agents is strongly influenced by abiotic factors, such as temperature, humidity, and exposure to UV radiation, as well as interactions with native microbiota. Consequently, current research focuses on strain selection, formulation technologies, and elucidation of the molecular mechanisms underlying antagonism to improve their stability, efficacy, and field applicability [20,21,22].

### 2.2. Yeast Biocontrol Agents

Yeasts are another promising group of biological control agents owing to their ecological versatility, rapid colonization capacity, and recognized safety. Their effectiveness is largely attributed to their strong competitiveness for nutrients and space on plant surfaces, especially in wounds and other areas of infection, where pathogenic fungi often begin colonization. Yeasts are also widely applied in above-ground (epiphytic) contexts within sustainable agriculture, where they function as effective biocontrol agents and biostimulants. They rapidly colonize plant surfaces—including leaves, fruits, and flowers—competing with pathogens for nutrients and space while simultaneously enhancing plant immune responses [23]. Yeasts are an important group of biological control agents for the management of fungal diseases in a wide range of crops, including cereals, legumes, oilseed crops (sunflower and canola), and horticultural crops [24].

Several yeast genera, including *Candida*, *Metschnikowia*, *Pichia*, *Aureobasidium*, and *Rhodotorula*, have demonstrated strong antagonistic activity against a wide range of phytopathogenic fungi. For example, Metschnikowia pulcherrima produces pulcherrimin, an iron-chelating pigment that restricts pathogen growth by limiting essential micronutrients [25]. Other species, such as *Pichia guilliermondii* and *Aureobasidium pullulans*, exhibit multiple mechanisms of action, including the secretion of lytic enzymes, biofilm formation, and induction of host defense responses [26].

**Figure 2:**
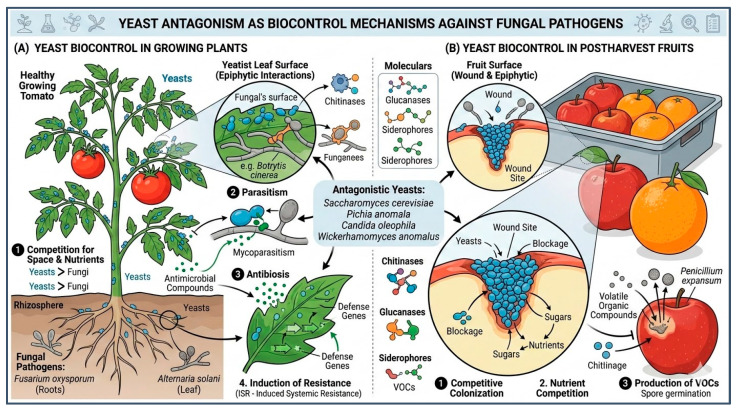
Schematic representation of yeast species with documented antagonistic activity as biocontrol agents against fungal pathogens in plants and post-harvest fruits. (**A**) represent effect of yeast in crops plants. (**B**) indicate how yeast can be used during postharvest.

Yeasts also show high tolerance to environmental stresses, such as low temperatures, osmotic fluctuations, and desiccation, making them particularly suitable for managing post-harvest diseases in fruits and vegetables. Their ability to survive and proliferate on plant surfaces improves persistence and contributes to long-lasting protection (Figure 2).

However, the performance of yeasts as biocontrol agents can be affected by factors such as nutrient availability, interactions with native microbiota, and physicochemical properties of the fruit surface. Current research seeks to improve formulation technologies, unravel the molecular mechanisms of antagonism, and explore synergistic combinations with other biological or natural compounds to improve their stability and efficacy under commercial conditions [27].

### 2.3. Bacterial Biocontrol Agents

**Figure 3:**
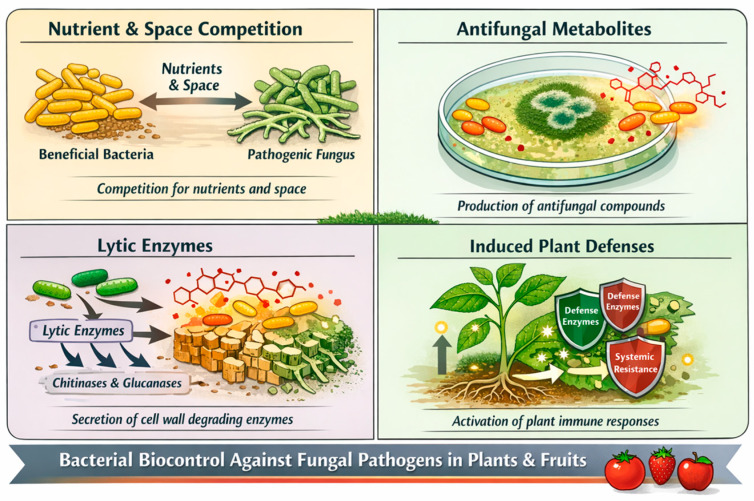
Schematic representation of bacterial species exhibiting antagonistic activity through mechanisms such as nutrient and space competition, production of antifungal metabolites, secretion of lytic enzymes, and induction of plant defense responses, functioning as biocontrol agents against fungal pathogens in plants and post-harvest fruits. Arrow with yellow circle indicate increase of plant immune responses.

Bacterial biocontrol agents (BCAs) are among the most studied and widely used microbial antagonists owing to their metabolic versatility, rapid proliferation, and ability to colonize various plant environments, including the rhizosphere, phyllosphere, and post-harvest environments. Their antagonistic activity against phytopathogenic fungi involves multiple processes, such as the synthesis of antimicrobial metabolites, competition for nutrients and different niches, development of universal resistance in plants, and secretion of lytic enzymes capable of degrading fungal cell walls [28] (Figure 3). BCAs play a central role in the sustainable management of fungal diseases affecting field crops, such as wheat, maize, rice, soybean, and sunflower [29].

Among the bacterial genera, *Bacillus* and *Pseudomonas* are the most widely studied. Bacillus species, particularly *B. subtilis*, *B. amyloliquefaciens*, and *B. velezensis*, are notable for their ability to produce endospores, which confer exceptional stability and persistence in adverse environmental conditions. These species synthesize a broad spectrum of bioactive lipopeptides, such as surfactin, iturin, and fengicin [28,30], which possess potent antifungal properties and alter the membrane integrity of pathogens. Their robustness and ease of formulation have facilitated their incorporation into commercial products [31].

*Pseudomonas* species, particularly *P. fluorescens* and *P. putida*, produce siderophores, antibiotics, and volatile organic compounds that inhibit fungal growth. Many strains can also trigger universal inducible defense in plants, increasing their ability to protect themselves against subsequent pathogen attacks. Their adaptability to the rhizosphere and ability to modulate plant-microbe interactions make them valuable components of integrated disease management strategies [32,33,34].

Other bacterial genera, such as *Streptomyces*, *Paenibacillus*, and *Burkholderia*, have also demonstrated significant biocontrol potential through various biochemical pathways and ecological interactions. However, the performance of bacterial antagonists is affected by environmental variability, microbial competition, and formulation challenges. Current research focuses on optimizing strain selection, improving formulation technologies, and elucidating the molecular mechanisms of antagonism to increase their reliability and efficacy under field and post-harvest conditions [28].

Despite recent progress, current biocontrol strategies still face important limitations due to the inconsistent performance of antagonistic agents under fluctuating environmental conditions. These constraints highlight the need to develop new biocontrol approaches that are more efficient, robust, and stable, which remains a critical priority for the effective management of fungal diseases in plants [8]. Moreover, the implementation of BCAs gives rise to a complex array of legal challenges. At their core, these challenges emerge from the intricate interplay between trade laws, environmental policies, and international relations.

### 2.4. Viruses as Biocontrol Agents

**Figure 4:**
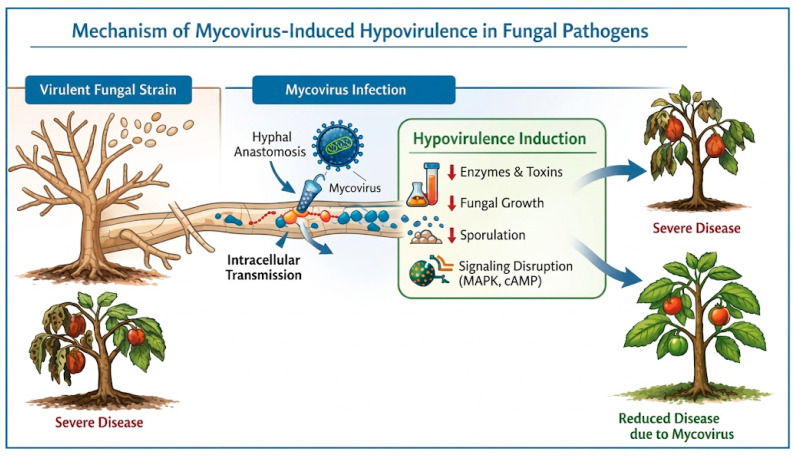
Illustration of the mechanisms of viruses as biocontrol agents against fungal pathogens in plants and post-harvest fruits. Red arrows show how hypovirulence is induced. Blue arrow showed how from severe disease, mycoviruses can reduce disease.

The use of viruses as biocontrol agents against fungal phytopathogens has emerged as a promising strategy within integrated disease management, offering high specificity, environmental safety, and long-term mycoviruses to induce hypovirulence, which is defined as a significant reduction in the virulence of the fungal host. This phenomenon has positioned mycoviruses as potential biological alternatives to chemical fungicides [35]. Mycoviruses exhibit substantial genetic and structural diversity and are transmitted exclusively through intracellular mechanisms, including hyphal anastomosis, cell division, and vertical transmission via spores in some species. This restricted mode of transmission contributes to their host specificity and environmental safety but also limits their natural dispersal [36] (Figure 4).

Hypovirulence induced by mycoviruses can arise through multiple physiological and molecular mechanisms, such as the alteration of fungal metabolism, including reduced production of cell walls. The most successful and well-studied Cryphonectria *hypovirus* 1 (CHV1) has been used to manage chestnut blight caused by *Cryphonectria parasitica*. Other notable examples include *Rosellinia necatrix megabirnavirus 1*, which reduces virulence in *R. necatrix*, and *Sclerotinia sclerotiorum* hypovirulence, which is distinguished by its ability to transmit extracellularly, thereby facilitating field application [37,38]. However, significant limitations remain. These include restricted horizontal transmission in fungi with strong vegetative incompatibility systems, the variable environmental stability of viral particles, and the need for controlled inoculation strategies to ensure successful establishment in the field. However, promising research directions include engineering viruses with enhanced transmission capacities and discovering novel hypovirulence [39].

Recent studies have shown that transcription factors regulating secondary metabolites can strengthen biocontrol of plant defense. Current studies highlight that AP2/ERF, WRKY, bHLH, bZIP, MYB, and NAC transcription factors are key regulators of secondary metabolite biosynthesis under biotic stress. These metabolites, including phenolics, terpenoids, and alkaloids, are essential for plant immunity and can synergize with biocontrol agents by reinforcing plant barriers and antimicrobial activity [40]. Moreover, plant growth has been studied [41,42]. TF networks orchestrating pathogen families such as WRKY, ERF, MYB, NAC, and bZIP act as master regulators of plant immune responses, coordinating basal immunity, effector, and crosstalk with phytohormones. Understanding these networks provides targets for improving biocontrol compatibility and boosting plant resilience [43].

### 2.5. Plant Bioactive Compounds

In addition to microbial antagonists, plants generate a variety of bioactive compounds that play essential roles in the defense against pathogenic fungi and other biotic stress factors [44,45]. These molecules, classified as secondary metabolites, are not necessary for basic metabolic functions but are crucial for the adaptation, communication, and survival of plants under pathogen pressure. Their biological activity, structural diversity, and ecological relevance make them important components of integrated disease management strategies and valuable complements to microbial biocontrol agents [46].

The bioactive compounds in plants can be broadly grouped into phenolics, terpenoids, alkaloids, sulfur-containing compounds, and bioactive peptides or proteins, each of which contributes to defense through different biochemical and molecular mechanisms [47]. Phenolic compounds, such as flavonoids, phenolic acids, and tannins, exert antioxidant, antimicrobial, and structural reinforcement functions and often concentrate rapidly at sites of infection [48]. Terpenoids, including monoterpenes, sesquiterpenes, and diterpenes, act as volatile signals and potent antifungal agents capable of disrupting pathogen membranes and inhibiting spore germination [45,49,50]. Alkaloids, characterized by their nitrogen-containing structures, exhibit strong antimicrobial activity and frequently participate in inducible defense responses [51].

Sulphur-containing compounds, particularly glucosinolates and their hydrolysis products (e.g., isothiocyanates), exhibit broad-spectrum antimicrobial activity and are increasingly being studied as potential natural alternatives to synthetic fungicides [52]. In addition, plants produce various bioactive peptides and proteins, such as defensins, lectins, protease inhibitors, and cell wall-degrading enzymes (e.g., chitinases and glucanases), which directly inhibit fungal growth or act as signaling molecules in systemic resistance pathways [53,54,55].

**Figure 5:**
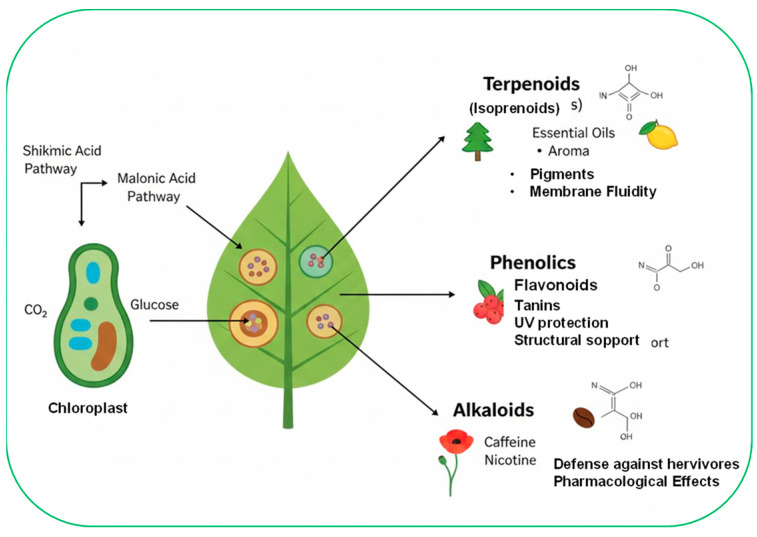
Schematic summary of bioactive plant compounds that contribute to plant defense through direct mechanisms, such as terpenoids, phenolic compounds, and alkaloids. Arrows indicate all products that can be obtained from plants.

In general, these compounds contribute to plant defense through direct mechanisms, such as inhibiting fungal growth, altering membrane integrity, chelating essential nutrients, and interfering with the enzymatic activity of pathogens. They can participate in indirect defense as key regulators of signaling pathways, such as salicylic acid, jasmonic acid, and ethylene, which orchestrate local and universal immune responses (Figure 5) [56].

Bioactive compounds in plants are particularly relevant, as many fruits and vegetables naturally increase the production of phenolic compounds, terpenoids, and phytoalexins after injury or pathogen attack. Their presence can act synergistically with microbial antagonists, thereby improving the overall effectiveness of biological control strategies [57]. Compounds such as resveratrol in grapes [58], escoparon in citrus fruits [59], and capsaicinoids in peppers exemplify the role of endogenous metabolites in limiting pathogen development during storage and handling [60].

Plant bioactive compounds face major obstacles to commercialization and therapeutic application due to their low stability, poor bioavailability, and limited solubility in water. These molecules are readily degraded by heat, light, oxygen, and fluctuations in pH, which significantly diminishes their efficacy. In addition, high extraction costs, environmental impact associated with solvent use, and variability in the quality of raw plant materials hinder consistent and scalable production of these compounds.

## 3. CRISPR-Based Genome Editing Diagnostics

**Figure 6:**
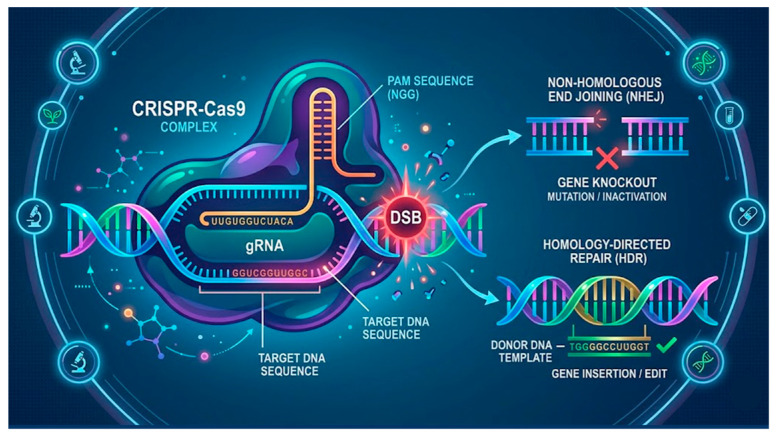
Schematic illustration of genome editing platforms (CRISPR) used to improve the diagnosis and control of fungal pathogens in plants. Red 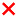 indicate interruption of the gene and 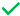 shows confirmation od insertion gene edit.

Innovations, such as genome-editing platforms, are reshaping the landscape of fungal pathogen control in modern agriculture. These technologies enable a more precise understanding of the biology of plant-pathogenic fungi, improve the early detection of infections, and facilitate the development of crops with more robust genetic resistance [61] (Figure 6).

The emergence of CRISPR-based genome-editing technologies has opened new avenues for understanding and controlling plant fungal pathogens with unprecedented precision. Unlike traditional approaches, CRISPR systems enable specific modifications with single-nucleotide resolution, thereby allowing the analysis of host–pathogen interactions and the more efficient design of disease-resistant crops. These advances are transforming modern plant pathology and offer sustainable alternatives to chemical fungicides [62].

CRISPR-Cas systems have been widely applied to modify plant genes associated with susceptibility and immunity. By inactivating susceptibility (S) genes or enhancing the expression of resistance (R) genes, researchers have successfully generated crops with greater resistance to major fungal pathogens [63]. This strategy is particularly effective because it avoids the introduction of foreign DNA, enabling the development of non-transgenic resistant varieties that comply with evolving regulatory frameworks and are publicly accepted. CRISPR has also become a powerful tool for studying the mechanisms of fungal virulence. Genome editing in fungi allows for the selective disruption of effector genes, signaling pathways, and metabolic processes essential for infection [64].

Ethylene Response Factor (ERF) transcription factors are central regulators of plant growth and stress resilience. ERFs direct the spatial allocation of resources and enable temporal alternation between growth and defense states. Emerging technologies, such as CRISPR-based genome editing, can leverage ERF regulatory networks, offering promising strategies for developing crops with precisely tuned adaptability, thus enabling sustainable agriculture even under changing climatic conditions [42].

## 4. RNA Interference (RNAi)

RNA interference (RNAi) has become an important and highly specific strategy for controlling fungal pathogens in plants. It consists of a conserved eukaryotic regulatory mechanism based on small RNA molecules, mainly small interfering RNAs (siRNAs), that leads to the deprivation or silencing of specific sequences of target mRNAs. In plant disease control, RNAi enables the targeted suppression of essential fungal genes involved in pathogenicity, development, and survival, offering a precise and environmentally sustainable alternative to conventional fungicides [65,66].

**Figure 7:**
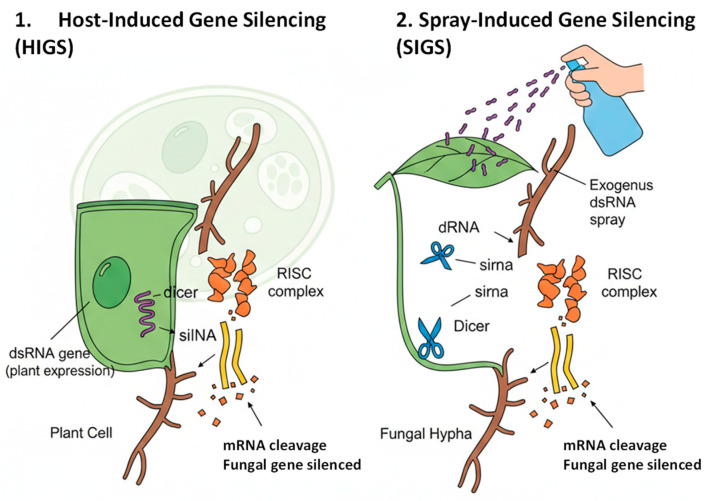
Outline of two main RNA interference-based procedures for plant control: host-induced gene silencing (HIGS) and spray-induced gene silencing (SIGS).

Currently, there are two RNAi-based approaches for plant protection: host-induced gene silencing (HIGS) and spray-induced gene silencing (SIGS). HIGS involves the transgenic expression of double-stranded RNA (dsRNA) in the host plant, which is subsequently taken up by invading fungi, causing the silencing of pathogen genes that are critical for infection [61]. This strategy has demonstrated strong efficacy against several fungal pathogens, including *Fusarium*, *Botrytis*, *Puccinia*, and *Colletotrichum*, by targeting genes associated with toxin biosynthesis, cell wall remodeling, and effector secretion [67,68]. HIGS provides long-lasting protection but requires the genetic modification of the host, which limits its regulatory acceptance and applicability in certain crops and markets [69]. In contrast, SIGS is based on the exogenous application of dsRNA or siRNA molecules to the plant surface. These RNA molecules can be directly absorbed by fungal spores or hyphae, triggering gene silencing without the need for transgenic plants. SIGS has gained importance because of its flexibility, safety, and compatibility with integrated pest management programs (Figure 7) [70]. Studies have shown that externally applied dsRNA can effectively suppress fungal growth and virulence by acting on genes involved in ergosterol biosynthesis, signal transduction, and detoxification pathways [71,72]. In addition, SIGS can be combined with microbial antagonists or natural compounds to improve their stability and absorption, thereby enhancing their field performance.

The success of RNAi-based control depends on several factors, such as the efficiency of dsRNA uptake by the pathogen, the stability of RNA molecules under environmental conditions, and the selection of appropriate genetic targets. Advances in nanocarrier technologies, such as clay nanosheets, liposomes, and polymeric nanoparticles, have improved the protection of dsRNA against UV degradation and optimized its delivery to fungal cells. Furthermore, the discovery of RNA trafficking between kingdoms has revealed that plants, fungi, and even beneficial microbes can exchange sRNAs, opening new avenues for the design of synergistic biocontrol strategies [73].

RNA interference (RNAi) faces several critical challenges, particularly those related to off-target effects, efficient in vivo delivery, and unwanted activation of the immune system. While RNAi has great potential, its translation into control effects is hampered by the rapid degradation of siRNA by nucleases, potential cytotoxicity, and transient gene silencing. RNAi-based fungal control faces challenges related to production costs, environmental persistence, and variability in the uptake among fungal taxa. Efforts are currently underway to optimize dsRNA design, improve formulation technologies, and elucidate the molecular mechanisms that govern RNA uptake and processing in fungi. As these limitations are resolved, RNAi will become a key component of next-generation sustainable plant disease management strategies [73,74].

However, RNAi-based technologies face several barriers to achieving broad market adoption. Regulatory frameworks remain fragmented, with countries applying different criteria for evaluating dsRNA products, particularly regarding environmental risks, off-target effects, and persistence. Approval pathways are still evolving, which slows product registration and increases uncertainty for developers [75].

On the commercialization side, high production costs, limited large-scale manufacturing capacity, and the need for robust delivery systems (to ensure stability and uptake in field conditions) remain major obstacles. The market is still emerging, with few registered products and uncertain returns on investment. Additionally, public perception and acceptance of RNA-based biotechnologies can influence adoption, requiring transparent communication and robust safety data [76].

Overall, RNAi technologies show high potential, but regulatory harmonization, cost-effective production, and validated field performance are essential for successful commercialization [77].

## 5. Nanotechnology for Fungal Disease Management

**Figure 8:**
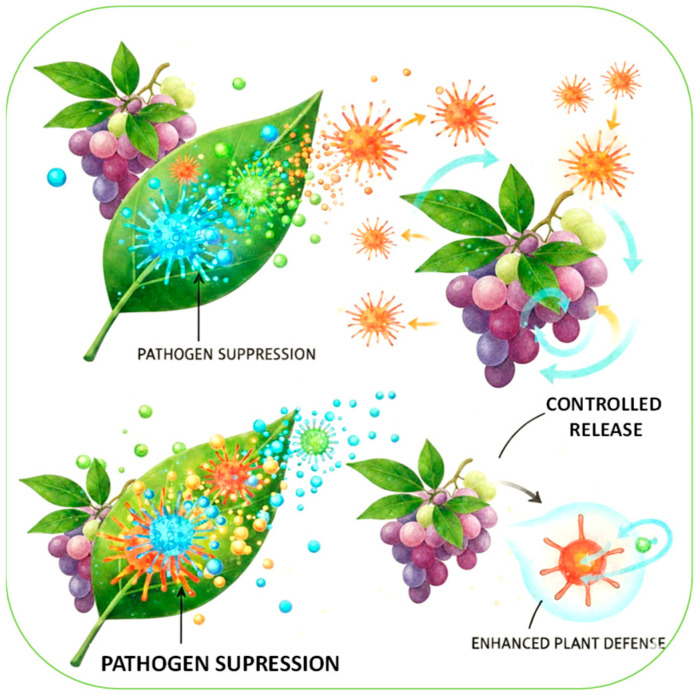
Nanotechnology is a powerful and versatile platform for the control of fungal diseases in agriculture through pathogen suppression or resistance induction.

Nanotechnology is another influential and versatile tool for managing fungal diseases in agriculture and post-harvest systems. Its relevance lies in the unique properties of nanomaterials (high surface-to-volume ratio, adjustable reactivity, and the ability to design controlled or targeted delivery), which, taken together, overcome many of the limitations associated with conventional fungicides and antifungals (Figure 8) [78]. The ability to load nanoparticles with multiple fungicidal agents exemplifies a strategy that not only improves treatment efficacy but also mitigates the risk of resistance development among fungal populations [79].

Recent studies show that nanoparticles of metals and metal oxides, such as silver, copper, zinc oxide, and iron oxide, possess strong antifungal activity through processes such as the generation of reactive oxygen species, membrane disruption, and interference with mitochondrial and genomic integrity [80,81]. An example of this has been carried out to inhibit *Penicillium expansum*, where natural phenol-Cu2-xSe nanoparticles with photothermal conversion capacity were developed. The antifungal activity was attributed to the structural alteration of the fungal cell walls and membranes, as well as mitochondrial dysfunction. This demonstrates the potential of photothermal conversion nanomaterials and their combination with NIR irradiation as a novel strategy for fruit preservation [82]. On the other hand, biopolymer-based nanocarriers, particularly those formulated with chitosan, PLGA, or alginate, allow the encapsulation of synthetic fungicides, essential oils, peptides, or natural antifungal compounds, improving solubility, stability, and release kinetics while reducing toxicity [83]. In addition, lipid-based systems such as nanoemulsions, liposomes, and nanostructured lipid carriers improve the delivery of hydrophobic or volatile antifungal agents and show great potential for penetrating biofilms and improving field performance [84]. Carbon composite nanomaterials, graphene oxide, carbon nanotubes, and carbon quantum dots also exhibit antifungal effects, although their environmental fate and biosafety require further study [85].

In crop protection, nanoformulated fungicides improve adhesion, UV stability, and controlled release, increasing efficacy in field conditions while reducing application frequency and chemical load. Post-harvest systems benefit from nanocoatings, nanoemulsions, and edible films incorporating antifungal nanoparticles, which extend shelf life and reduce waste [86].

Nanotechnology presents significant challenges related to environmental toxicity, human health risks, and ethical considerations, primarily because nanoscale materials often exhibit unpredictable behavior compared to their macroscopic counterparts. Nanotechnology faces significant regulatory and commercialization challenges due to fragmented global standards, incomplete toxicological information, and evolving risk assessment frameworks [87]. Key concerns include the ability of nanoparticles to penetrate cell membranes, high production costs, difficulties in scaling up manufacturing, and complex intellectual property frameworks, which further hinder market entry. Public perception issues and a lack of harmonized international guidelines add further layers of uncertainty. Collectively, these factors slow the development, approval, and widespread adoption of nanotechnology-based products [88]. Nevertheless, advances such as stimulus-responsive nanocarriers, nanotechnology-enabled RNAi/CRISPR delivery, green nanoparticle synthesis, and integration with omics-driven precision agriculture point towards increasingly sustainable and multifunctional nanostrategies for fungal disease management [89].

## 6. Aptamers

**Figure 9:**
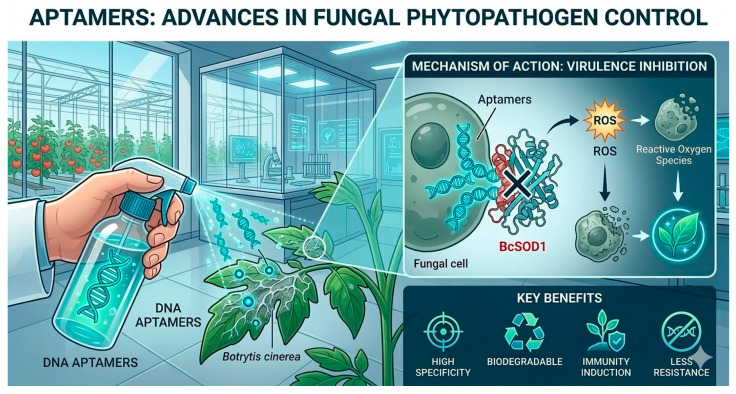
Graphical representation of aptamers adapted for the control of fungal pathogens in plants. The DNA aptamer-based strategy targets BcSOD1, a pivotal antioxidant enzyme in *Botrytis cinerea*, offering a highly specific and precise approach to suppressing fungal virulence. **X** indicate block of BcSOD1 gene and arrows (black and white) showed production of ROS and how they affect cell and therefore to the plant.

Aptamers are short nucleic acid sequences—composed of ribonucleic acid (RNA) or single-stranded deoxyribonucleic acid (ssDNA)—typically ranging from 25 to 80 bases in length. They are capable of binding selectively to specific target molecules, functioning in a manner comparable to monoclonal antibodies [90]. The remarkable adaptability of aptamers stems from their in vitro selection using SELEX (Systematic Evolution of Ligands by Exponential Enrichment), a process that allows the isolation of nucleic acid molecules capable of recognizing virtually any fungal target with high affinity. These targets can include enzymes essential for pathogen survival, virulence factors required for host colonization, structural components of the fungal cell wall, or secreted effectors that modulate plant immune responses. By designing aptamers to bind to these molecular determinants, it is possible to disrupt critical biological processes in a highly selective manner and with mechanistic precision. Aptamers offer a promising alternative for improving the detection and management of plant diseases, which are often limited by their sensitivity, specificity, and applicability in the field, thanks to their high affinity and selectivity [91]. Their robustness and versatility allow them to be used not only as sensitive biosensors for pathogen detection, but also as active molecules capable of interfering with pathogen effectors and modulating the immune response of plants. Although still an emerging technology, aptamers have great potential to transform future plant disease control strategies (Figure 9) [92].

Aptamers have emerged as a compelling alternative to antibodies due to their remarkable specificity and affinity, making them powerful tools for a wide range of detection applications. The integration of aptamer-based techniques with nanotechnology has further enhanced signal amplification in aptasensors, enabling faster diagnostics, reducing analytical costs, and improving overall accuracy [93].

A recent application of this DNA aptamer-based approach targets BcSOD1, a key antioxidant enzyme in *Botrytis cinerea*, providing a highly specific strategy for suppressing fungal virulence. By binding to and inhibiting *BcSOD1*, the aptamers compromise the pathogen’s defenses against oxidative stress and diminish its ability to colonize plant tissues. This technology offers a promising and sustainable alternative to conventional fungicides and enhances precision tools for the effective management of grey mold in agriculture [94].

Aptamer-based technologies face several regulatory and commercialization challenges that limit their widespread adoption in diagnostics, therapeutics, and biodetection. Regulatory frameworks remain less mature and standardized than those for antibodies, leading to uncertainty in aptamer approval processes. Commercialization is further constrained by manufacturing and scalability issues. While aptamers benefit from chemical synthesis, ensuring consistent folding, functional stability, and reproducibility at an industrial scale remains a challenge [95]. The intellectual property landscape is complex, which can increase licensing costs and restrict innovation. Market adoption is also slowed by limited knowledge among end users, competition with well-established antibody platforms, and the need for rigorous validation in real-world matrices [96].

## 7. Concluding Remarks and Future Perspectives

The growing pressure from fungal diseases, driven by climate change, the intensification of agricultural practices and the rapid emergence of fungicide resistance, highlights the urgent need for innovative and sustainable strategies for plant protection. This review highlights a clear shift away from reliance on conventional chemical fungicides towards a more diversified and integrated approach. Biological control agents remain crucial in this transition due to their ecological adaptability and multiple antagonistic mechanisms; however, their inconsistent performance under field conditions underscores the need to improve formulation, stabilization and application systems. Bioactive compounds of plant origin further expand the repertoire of natural antifungal defenses, acting both directly on pathogens and indirectly by modulating the plant’s immune response. At the same time, genome editing platforms, together with RNA interference technologies, offer unprecedented specificity for targeting fungal genes. Non-transgenic mechanisms such as spray-induced gene silencing have brought about an exciting revolution, although challenges related to environmental stability, absorption efficiency and production costs must be addressed to enable wider implementation. Nanotechnology is also emerging as a powerful enabling platform capable of improving the delivery, persistence, and efficacy of antifungal agents. However, its adoption requires rigorous assessment of environmental fate, biosafety, and regulatory frameworks. In addition, aptamers represent a promising class of precision molecular tools with potential applications in pathogen detection and targeted inhibition of virulence factors.

Looking ahead, it will be essential to strengthen the robustness of biological control agents through advanced strain selection, optimized formulations and improved ecological compatibility. Harnessing plant metabolic pathways through metabolic engineering or elicitor-based strategies may enable the controlled enhancement of endogenous defenses. Continued progress in RNAi technologies will depend on the development of stable and cost-effective delivery systems capable of ensuring efficient uptake of dsRNA in diverse fungal taxa. The integration of nanomaterials represents a multifaceted and highly targeted strategy for suppressing fungal pathogens in plants.

The design of safe and environmentally responsible nanomaterials, supported by comprehensive ecotoxicological assessments, will be critical for their responsible implementation. The expansion of aptamer-based platforms for practical diagnosis and targeted control could further advance precise disease management. Ultimately, the integration of these tools into holistic, crop-specific disease management programs will be key to achieving durable and environmentally safe control of fungal pathogens.

## Data Availability

No new data were created or analyzed in this study. Data sharing is not applicable to this article.

## Abbreviations

The following abbreviations are used in this manuscript:

NGS	Next-Generation Sequencing
CRISPR	Clustered Regularly Interspaced Short Palindromic Repeats
RNAi	RNA interference
siRNAs	small interfering RNAs
HIGS	host-induced gene silencing
SIGS	spray-induced gene silencing
dsRNA	double-stranded RNA
TF	Transcription factor
PLGA	Poly Lactic-co-Glycolic Acid
